# Infanticide in a mammal-eating killer whale population

**DOI:** 10.1038/s41598-018-22714-x

**Published:** 2018-03-20

**Authors:** Jared R. Towers, Muriel J. Hallé, Helena K. Symonds, Gary J. Sutton, Alexandra B. Morton, Paul Spong, James P. Borrowman, John K. B. Ford

**Affiliations:** 1 Pacific Biological Station, Fisheries and Oceans Canada, 3190 Hammond Bay Road, Nanaimo, BC V9T 6N7 Canada; 2 Bay Cetology, Box 554, Alert Bay, BC V0N 1A0 Canada; 3 OrcaLab, Pacific Orca Society, Box 510, Alert Bay, BC V0N 1A0 Canada; 4 Raincoast Research Society, Box 399, Sointula, BC V0N 3E0 Canada; 5 Whale Interpretive Centre, Box 2–3, Telegraph Cove, BC V0N 3J0 Canada

**Keywords:** Behavioural ecology, Conservation biology, Cultural evolution, Sexual selection

## Abstract

Infanticide can be an extreme result of sexual conflict that drives selection in species in which it occurs. It is a rarely observed behaviour but some evidence for its occurrence in cetaceans exists in three species of dolphin. Here we describe observations of an adult male killer whale (*Orcinus orca*) and his post-reproductive mother killing a neonate belonging to an unrelated female from the same population in the North Pacific. This is the first account of infanticide reported in killer whales and the only case committed jointly by an adult male and his mother outside of humans. Consistent with findings in other social mammals, we suggest that infanticide is a sexually selected behaviour in killer whales that could provide subsequent mating opportunities for the infanticidal male and thereby provide inclusive fitness benefits for his mother.

## Introduction

Conspecific infanticide occurs within many taxa. Among terrestrial mammals, it is mostly reported in primates, carnivores and rodents^1^. However, compelling evidence for infanticide in cetaceans exists in three species of dolphin. Patterson *et al*.^2^ and Dunn *et al*.^3^ found that dead neonate and juvenile common bottlenose dolphins (*Tursiops truncatus)* in the eastern and western North Atlantic respectively had scarring that suggested they were killed by conspecifics. More recently, Kaplan *et al*.^4^, Robinson^5^, Perrtree *et al*.^6^ and Díaz López *et al*.^7^ witnessed adult common bottlenose dolphins attack young in the North Atlantic. Furthermore, Zheng *et al*.^8^ documented adult Indo-Pacific humpback dolphins (*Sousa chinensis*) killing neonates in the western North Pacific and in the South Atlantic, an attack on a neonate Guiana dolphin (*Sotalia guianensis*) conducted by adults likely resulted in its death^9^.

In many cases, it is difficult to put the causes and effects of infanticidal acts into context because the natural occurrence of this behaviour is so rarely observed^10,11^. However, infanticide occurs in a myriad of different situations and several hypotheses have been proposed to explain it^11–13^. Salient among these is the sexual selection hypothesis which states that infanticide can benefit the fitness of males that commit it because loss of a dependent offspring ends lactational amenorrhea and can quickly return the mother to a fertile condition. In these cases, infanticide not only creates a mating opportunity but can also remove the progeny of a competing male from the gene pool^12^. The predation hypothesis suggests that infanticide precedes cannibalism and is more likely to take place in energy-stressed populations^13,14^. However, cannibalism does appear to be a secondary benefit of some infanticide events best explained by the sexual selection hypothesis^15,16^. The resource competition hypothesis predicts that the removal of an infant may provide greater access to prey, mates or habitat for the perpetrator or its descendants^12,13^. Lastly, non-adaptive explanations for infanticide purport that it is a socially pathological behaviour that may be conducted accidentally or as a result of environmental stressors^17,18^.

The risk and occurrence of infanticide is likely to have influenced social structures and mating systems in some species^11,19^. Although much research has been conducted on the social societies of cetaceans^20^, very little is known of the reproductive behaviour of most odontocetes^21^. However, male Indo-Pacific bottlenose dolphins (*Tursiops aduncus*) have been documented sexually coercing females by chasing, ramming and biting them^22^. These aggressive behaviours and incidents of newly acquired scars on females caused by the teeth of conspecifics are positively correlated with the timing of their reproductive cycles^23,24^. Additionally, while scars on several other odontocete species are thought to occur in mating competitions between males^25,26^, these intraspecific agonistic interactions are rarely observed. For example, although killer whales (*Orcinus orca*) are one of the most studied and widespread species of cetacean, only a few observations of aggression between individuals of the same population have been reported (see^27^). Nevertheless, individuals of all sex and age classes from many killer whale populations around the world have scars on their bodies from the teeth of conspecifics^28–33^. These scars may originate from rough interactions between or within sex and age classes but, causes of their occurrence likely vary in nature and among populations.

In the North Pacific Ocean several populations of both fish-eating and mammal-eating killer whale exist^34^. Among those that prey on marine mammals (Bigg’s killer whales) are a number of genetically distinct populations in the Gulf of Alaska^35^ as well as the West Coast Transient (WCT) population which is primarily found in coastal waters and along the continental shelf edge from central California to southeastern Alaska^36^. This population is currently considered Threatened in Canada^37^ but has been growing at an average rate of about 3% per year since 1975 and now consists of several hundred individuals^38,39^. Social units in this population usually contain two to six individuals and are typically composed of a reproductive female and her offspring. Both male and female offspring are known to permanently or temporarily disperse as they mature but post-reproductive females often travel with their adult sons continuously^40^.

Field studies on WCT killer whales conducted by researchers affiliated with the Pacific Biological Station in British Columbia, Canada began in 1973^41^. After more than 5300 photographically documented encounters with WCT killer whales, an encounter on 2 December 2016 provided the first direct observations of lethally agonistic behaviour between individuals in this population as an adult male and his mother killed a neonate belonging to a female that was not maternally related. The events leading up to and following the infanticide incident are presented chronologically below and the occurrence of this phenomenon within WCT killer whale society is discussed in context of the various hypotheses for this behaviour.

## Results

**Figure 1:**
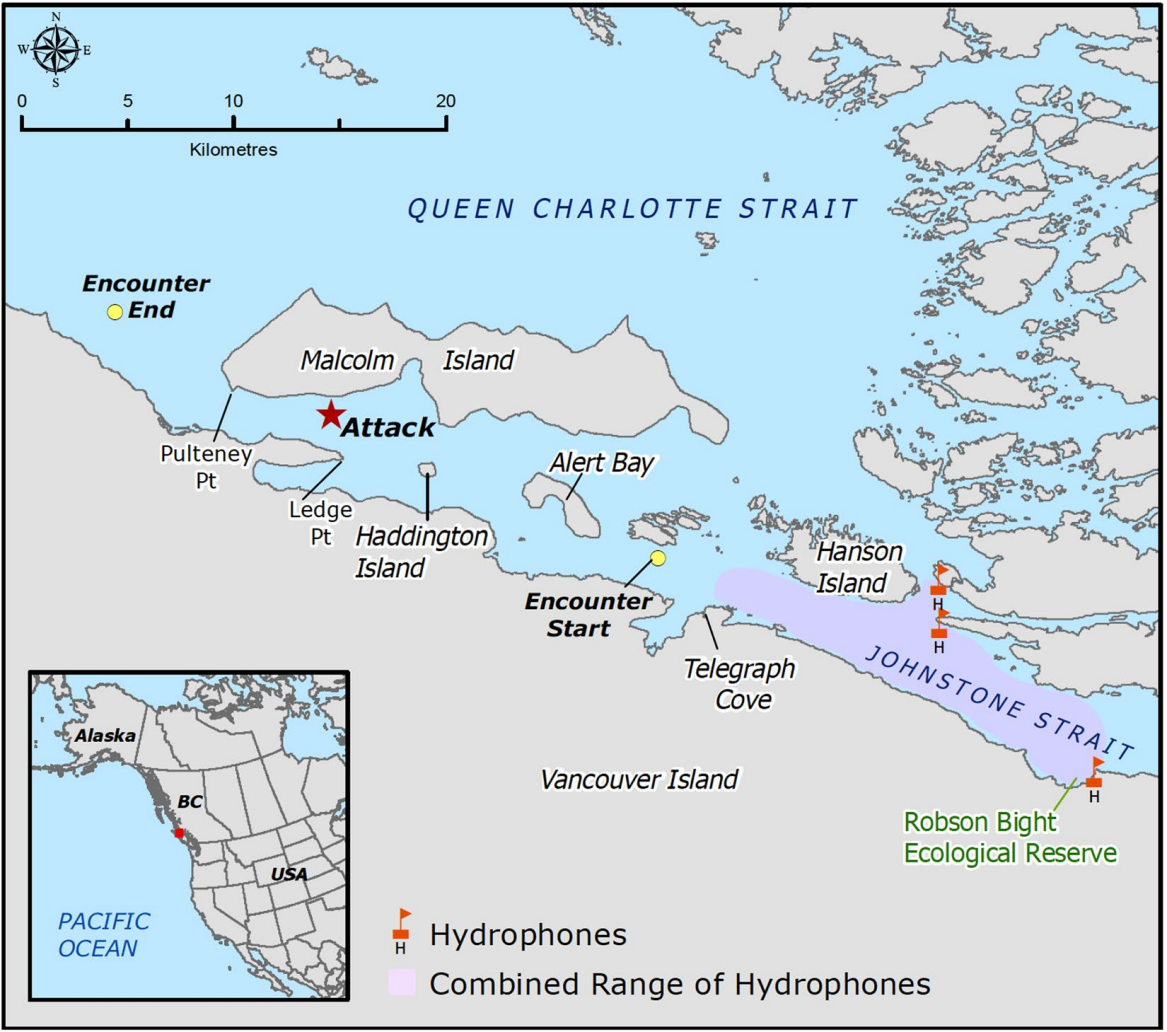
Map of study area (created with ArcGIS v10.5 - https://www.esri.com/en-us/home). The locations and acoustic range of hydrophone stations, place names, and positions of the encounter beginning, end and the infanticide event are provided.

**Figure 2:**
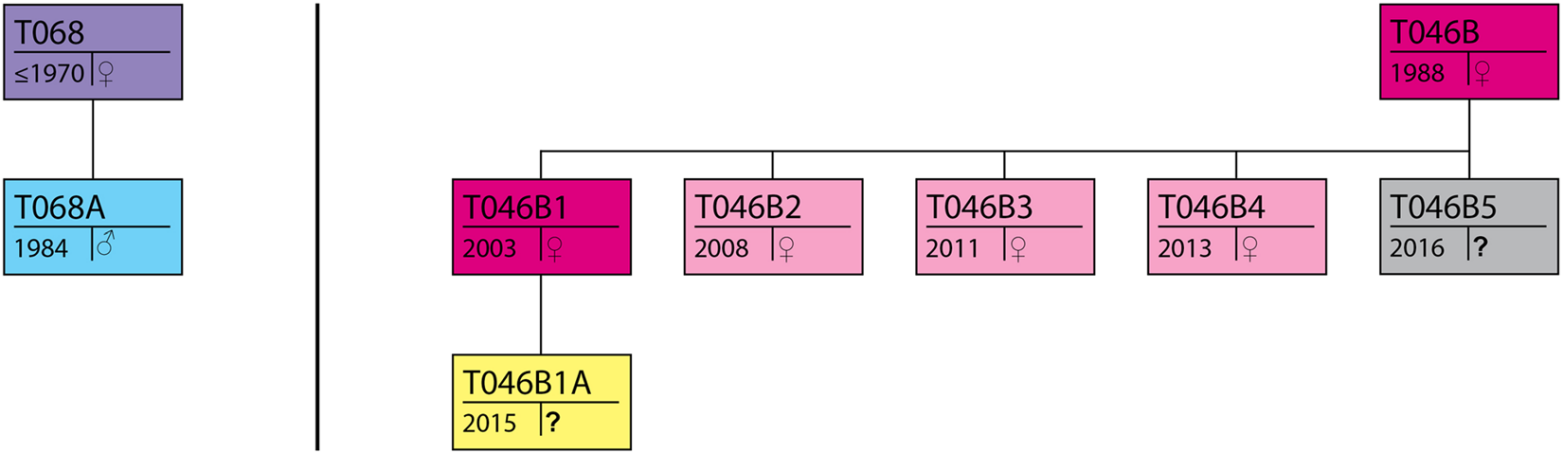
Genealogical schematic of present individuals. The identities and where known, sex and birth year of all whales present and their maternal relations to each other (see original schematics in Towers *et al*.^30^) displayed in colour coded statuses. Purple: post-reproductive female, Blue: reproductive male, Fuscia: reproductive females, Pink: juvenile females, Yellow: juvenile of unknown gender, Gray: neonate of unknown gender.

**Figure 3:**
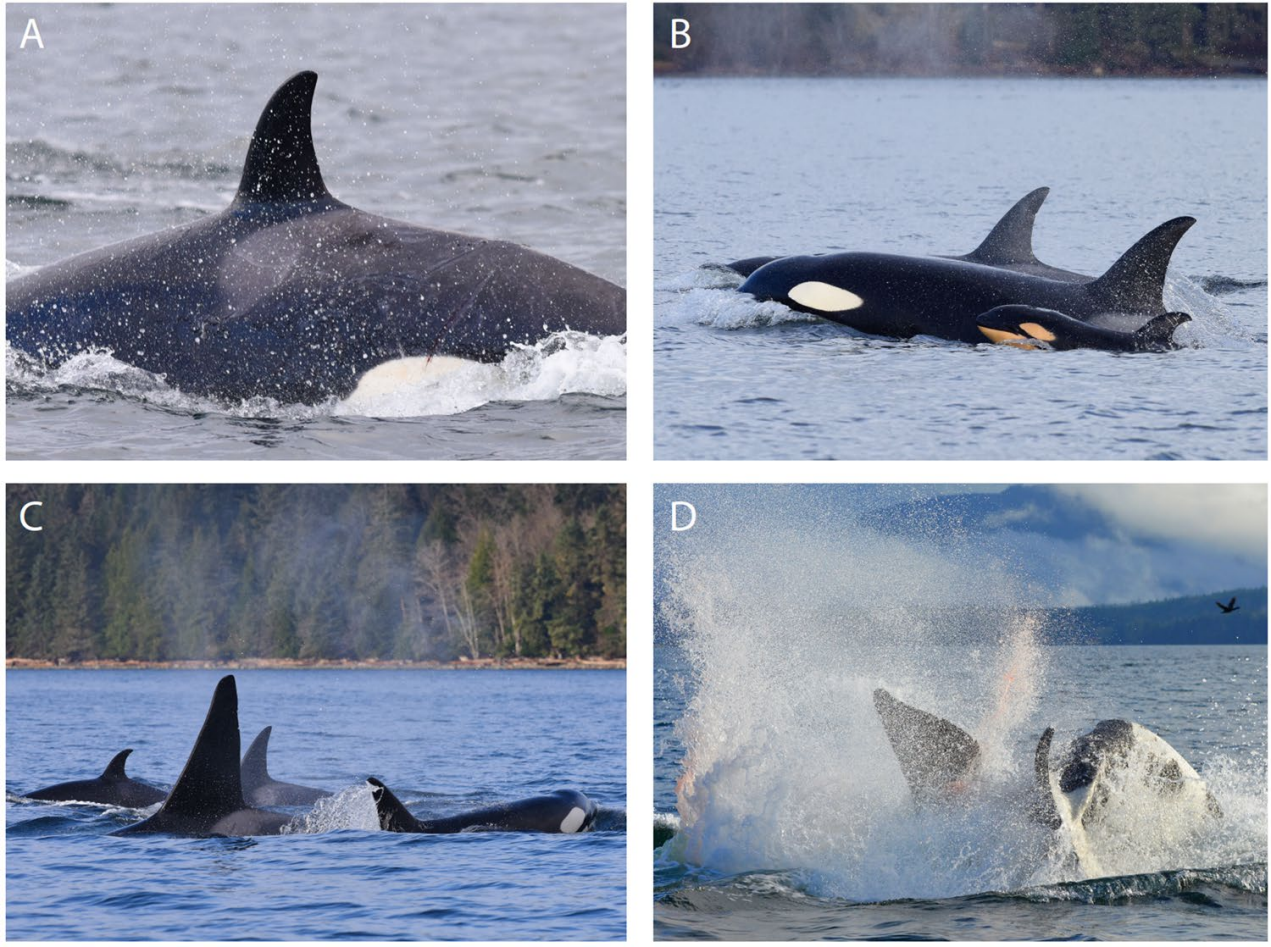
Observations leading to infanticide. (**A**) Fresh wounds on left flank and kinked spine anterior to dorsal fin on T046B4. (**B**) T046B with offspring T046B2 and (neonate) T046B5. (**C**) T068A surrounded by T046B, T046B1 and T046B1A. (**D**) T046B ramming T068A from below sending spray and blood into the air.

At 09:59 WCT killer whale vocalizations were detected on a remote hydrophone station located near Robson Bight in Johnstone Strait. By 10:10 vocalizations were also heard on two other hydrophone stations about ten km to the west, indicating a westward movement of the whales (Fig. 1). Between 10:06 and when the last vocalization was recorded at 11:01, two or more individuals repeated several discrete and aberrant pulsed calls (Supplementary Recording S1; Supplementary Table S1). At 10:55 a research boat was dispatched from Alert Bay to intercept the whales and they were spotted heading west in western Johnstone Strait at 11:07. Adult female T068 (age ≥ 46 yr, thus post-reproductive) and her adult son T068A (age 32) were travelling together about 200 m behind a young mother T046B1 (age 13), her offspring T046B1A (age < 2 yr) and her sister T046B4 (age < 3 yr) (Fig. 2). The three T046B individuals were traveling rapidly between 11 and 17 km/h. T046B4 had fresh wounds from the teeth of another whale on both flanks and a noticeable kink on the dorsal surface of the ridge posterior to the dorsal fin that was associated with broken skin (Fig. 3A). The wounds on the left flank were still bleeding (Fig. 3A). The T068s were keeping a steady pace behind and off to the side of the T046Bs. At 11:29, the mother of T046B1 and T046B4, T046B (age 28 yr), was found west of Alert Bay about a kilometer ahead of the other whales with her other daughters T046B2 (age 8 yr) and T046B3 (age 5 yr) and a neonate of unknown sex with visible fetal folds and a dorsal fin that was not yet entirely erect (T046B5) (Figs 2 and 3B). They remained ahead of the rest of the T046Bs until 11:55 when they all came together near Haddington Island. The T068s remained roughly 200 m behind. All whales were making a steady pace to the west.

Nearing Ledge Point at 12:18, a portable hydrophone was deployed ahead of the whales, but no vocalizations were heard. A few minutes later, after the whales had passed about 500 m to the west, erratic movements and splashing suggestive of a predation event were observed. Upon arriving on scene at 12:27, T068A was approximately 150 m from the other whales and moving away from them. The other whales followed. They all came together after 1–2 minutes and began circling vigorously (Fig. 3C). It was realized that the neonate T046B5 was not surfacing next to its mother T046B or in the vicinity and then T068A swam close past the research boat and the fluke of the neonate could be seen in his mouth with the body intact trailing underneath his lower jaw. Movements of all whales were erratic but the neonate’s mother T046B appeared to be chasing the male T068A while his mother, T068 attempted to manoeuvre between them (Supplementary Movie S1). Intense vocal activity could be heard through the hull of the boat so the hydrophone was deployed. A wide variety of excited discrete and aberrant pulsed calls (Supplementary Table S1), whistles, and percussive sounds were recorded (Supplementary Recording S2). Underwater video obtained during this time shows T068A with an object partially white in colour in his mouth, closely flanked by T068 (Supplementary Movie S2). At 12:35, T046B rammed T068A near the surface with sufficient force to cause a noticeable undulation through his body, sending blood and water into the air (Fig. 3D).

**Figure 4:**
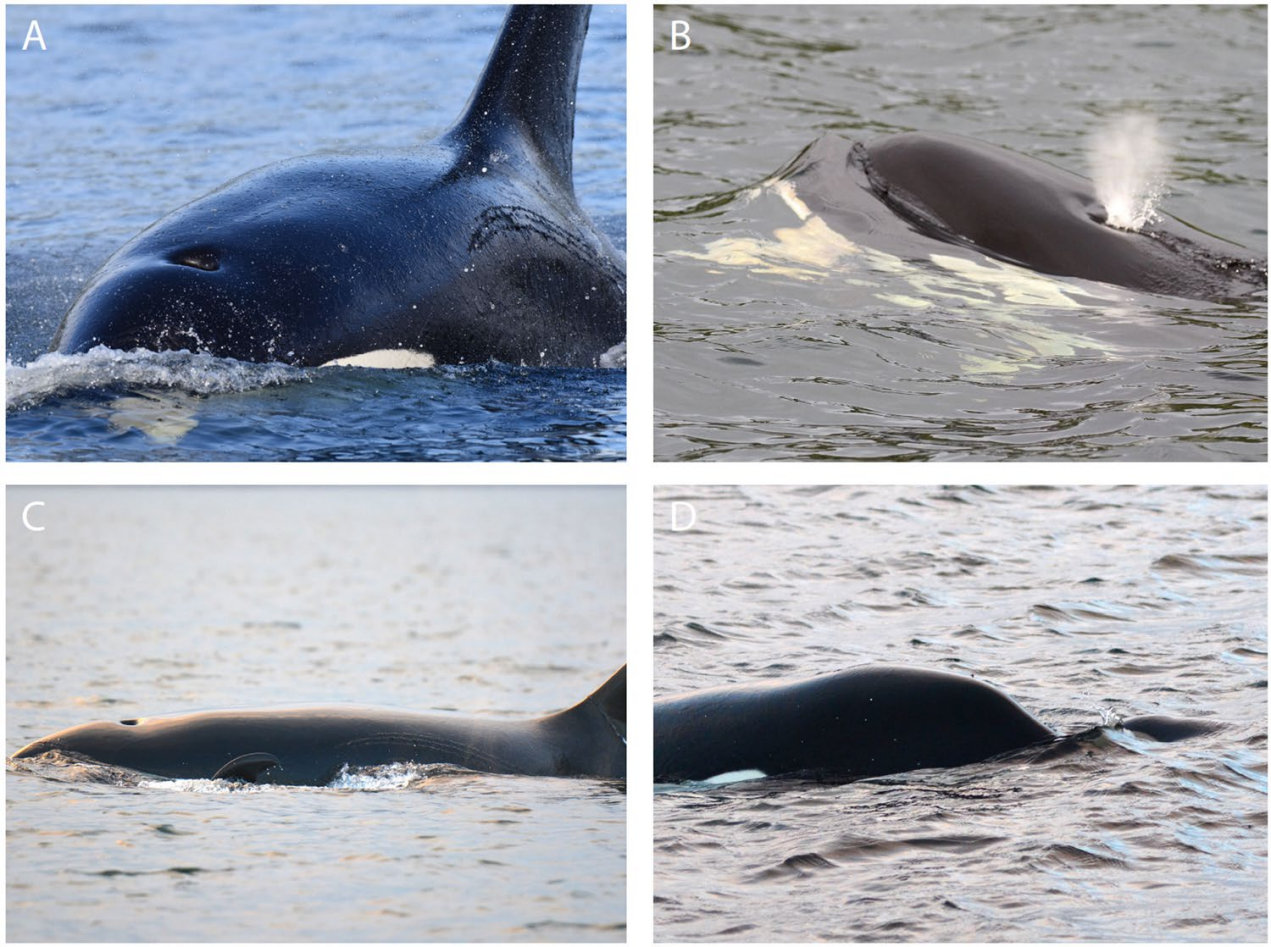
Observations following infanticide. (**A**) T068A with the fluke of T046B5 in the left side of his mouth. Fresh scars from the teeth of another whale can be seen on his left flank and rostrum. (**B**) T068 surfacing with the fluke of T046B5 visible in the left side of her mouth. (**C**) T068A surfacing with the dorsal fin of T046B5 visible off the left side of his melon. (**D**) T068 rostrum to rostrum with T046B5 approximately 215 minutes after its death.

At 12:43 the activity began to subside. Fresh teeth wounds that were not present before the incident were visible on T068A’s left and right flanks (Fig. 4A), rostrum, and base of dorsal fin and on T068’s melon. T068A and T068 began to withdraw from the others and slowly travel west. T068A still had the neonate by the fluke in his mouth (Fig. 4A). At 13:07, his mother T068 was filmed and photographed packing the neonate by the fluke (Supplementary Movie S3; Fig. 4B). The T046Bs remained 200–300 m behind and off to the side of the T068s, surfacing together and travelling slowly. At 13:42 all whales were off Pulteney Point and the dorsal fin of the neonate was seen above the surface on T068’s right side as she surfaced, indicating she was holding its left pectoral flipper in her mouth. At one point she pushed the neonate along in front of her and its head was visible above the surface before sinking back down into the water. A few minutes later the T046Bs slowly approached them from behind. T046B came out ahead of the others within about 50 m of the T068s but then reversed direction and re-joined her group. After this the T046Bs remained grouped up within a kilometre of the T068s for the duration of the encounter. At 14:20, underwater footage of the T068s showed neither of them to be holding T046B5, but just prior to this they had begun to circle at the surface after making long dives indicating that the neonate may have been below them. At 14:50 it was visually confirmed that the T046Bs were not in possession of the neonate. After not seeing T046B5 since before 14:00, T068A surfaced at 16:00 with its dorsal fin visible off the left side of his head (Fig. 4C), indicating he held its right pectoral flipper in his mouth. He was photographed surfacing rostrum to rostrum with the dead neonate a few minutes later (Fig. 4D). Its body appeared to be intact. After unsuccessfully attempting to acquire a biopsy sample of T068A, observations ended at 16:15 due to failing daylight. No evidence of feeding behaviour such as oil slicks or birds becoming interested in the whales were observed at any point.

## Discussion

WCT killer whales may be well adapted behaviourally for performing infanticide because of their experience killing other small cetaceans. Individuals typically dispatch these prey by ramming or crushing them with their rostrums or tails^36^. Observations of infanticidal behaviour towards young common bottlenose dolphins, Indo-Pacific humpback dolphins and a Guiana dolphin by conspecifics have included repeated ramming and crushing actions but also forced submergence^4–9^. Although we did not observe the first few minutes of this incident closely enough to determine the details of interactions that took place, it appeared as though T046B5 was quickly immobilized by having its tail gripped in the teeth of T068A and subsequently drowned because there was no opportunity to surface and breath due to his consistent forward motion. Although both T068 and T068A were observed with fresh teeth scars after the incident, the only ramming behaviour we observed was performed by T046B toward her neonate’s killer. This combative behaviour is similar to actions directed towards infanticidal males by the mothers of targeted infants in several rodent, primate and carnivore species (see review in^13^). The ramming occurred approximately ten minutes after the incident began, at which point the neonate would have been either close to or freshly drowned, which is probably why the fight did not persist. The brevity of this physical aggression might be typical of agonistic interactions between members of the WCT killer whale population. This, in addition to the fact that most exchanges between individuals take place below the surface, may help explain why none of this nature have previously been observed. Similarly, anomalistic calls are not commonly made by whales in this population^42^, but as many animals are known to produce atypical sounds under stressful situations^43^, the high number of aberrant and discrete pulsed calls and other excited sounds recorded before and during the infanticide event likely reflect the levels of intensity in the complex interactions taking place between individuals. For example, the fresh trauma observed on T046B4 at the beginning of this encounter indicates that other rough interaction between the whales occurred prior to our arrival. This may have caused the T046Bs to separate into two groups to benefit the neonate and the other young animals through their disassociation from it, but vocalizations between them and their ultimate re-grouping may have revealed their locations to the T068s.

The motivation for T068A and T068 to make this attack is of particular interest, but that they carried it out cooperatively is not surprising because bonds between maternally related killer whales can be particularly strong^34^. In sympatric populations, post-reproductive female killer whales increase the survival of adult sons by sharing ecological knowledge and prey with them^44,45^. This benefits inclusive fitness of the female because a positive relationship exists between reproductive success and age in male killer whales^45,46^. This combined with a prediction by Connor *et al*.^47^ that post-reproductive female killer whales may play a role in acquiring mates for their adult male offspring suggests that T068’s active involvement in this event was sexually selected, especially considering that T068A is of a reproductively mature age^30,46^. The sexual selection hypothesis requires that the infanticidal male does not kill his own offspring, that the event provides a near future mating opportunity with the infants’ mother and that he sires offspring with her^12^. Although it has been confirmed in many other mammal species that infanticidal males are not the fathers of their victims^48–55^ and that they subsequently sire offspring with the infants mothers^13,15,51,53,55–57^, in this incident the neonate’s carcass could not be recovered, T068A was not biopsied for DNA analysis and T046B has not yet had another calf.

However, further evidence also suggests that the infanticidal teamwork of T068 and T068A was motivated by potential to increase their inclusive fitness. For example, young offspring are often selected for infanticide because new mothers return to estrus much quicker than those that have been lactating for longer periods of time^12^. In this case, we know that T046B5 was less than a few days old and that suckling stimulates lactation in female killer whales whose postpartum return to estrus is much longer (5–32 months) when lactating than when not (1–4 months)^58^. Secondly, females in some animal societies copulate more with coercive males^59,60^ that restrict parental investment and improve the female condition by committing infanticide to increase their fitness^61^. In this case, although it is not clear if the attack on T046B4 was attempted juvenilicide, this whale (although third youngest in the group) was T046Bs second youngest offspring, so its death along with loss of her neonate could have benefitted her condition by reducing any prohibitive physical costs associated with caring for either of them. This would help restrict parental investment towards any forthcoming offspring of T068A if he successfully mated with T046B. Furthermore, infanticidal males are typically not familiar with the infant’s mothers^13^ and although unrelated matrilineal groups of WCT killer whales have often been known to spend several weeks together^40^ in what have generally been perceived to be mutual associations, T068A, T068 and T046B had only been documented together on two previous occasions – once in 2005 and again in 2007. The apparent low levels of association between these individuals suggest that they are not well known to each other and this may be reflective of inherent sexual conflict between their differing sex and age classes.

Sexually selected infanticide in WCT killer whales has important implications for our understanding of the social behaviour and evolution of this species (Supplementary Discussion S1) and although in this case, the hypothesis cannot be proven, there is more supporting evidence for it than other hypotheses for this behaviour. For example, WCT killer whales are well known for their dramatic hunting behaviour and protracted kills that typically result in immediate vocal and social activity, including division and consumption of mammalian prey^36,62^. However, in this incident the vocal and social activity subsided once the kill was made and although the dead neonate was kept in the possession of its killers for at least 220 minutes, there was no indication that it was dismembered or consumed. Additionally, most prey species that WCT killer whales target have been steadily increasing in coastal waters over the last few decades^63–69^. These increases in prey availability have been commensurate with increasing social activity^70^ and higher recruitment within the WCT population^39^ suggesting that it is not nutritionally stressed. Thus, this incident is not easily explained by the predation hypothesis. Furthermore, although the WCT population has more than doubled in size since 1990^39^, there is little evidence available to suggest that individuals within it compete for habitat or prey^71^. Regardless, these and the availability of other resources such as mates would not easily be threatened by an infant of either sex due to the slow rate of maturation in this species. Thus, this incident is not easily explained by the resource competition hypothesis. Finally, there is little support for interpreting infanticide as a non-adaptive behaviour other than when it occurs by accident or under unnatural conditions^13,53,72^ and contrary to accidental or pathological explanations, the infanticidal behaviour of T068A and T068 appeared goal oriented because their chase led to an attack that was maintained until a particular outcome had been achieved. Once it had, their behaviour immediately changed.

In conclusion, given that infanticide is so rarely observed in terrestrial mammals it is not surprising that it has taken many years of directed field observations to confirm that it does occur in cetaceans and more specifically, in killer whales. This species shares many life history traits with other mammals that are known to commit infanticide (see^1^), such as a high lactation to gestation ratio^58^, a society where individuals live in stable mixed sex groups where calves are born year round^71^ and a breeding system that is monopolized by a minority of mature males^46^. As a result, this phenomenon might be expected to occur in more killer whale populations than just the WCT. In any case, additional study is required to investigate any impacts that this phenomenon may have on the evolution of the social structure of this population and the behaviour of individuals within it.

## Methods

Underwater acoustic data were transmitted live from three fixed hydrophone stations maintained by OrcaLab in or adjacent to Johnstone Strait to monitors in Alert Bay, Telegraph Cove and on Malcolm Island. Vocalizations were digitally recorded and classified through visual and aural comparison using Audacity spectrograms and call type catalogues for this population provided in Ford^73^ and Deecke^62^. Calls were considered aberrant if they were similar but not identical to stereotyped calls. Vocalizations that were impossible to identify due to signal strength and/or interference were classified as unidentified. Digital audio, video and images were obtained in the field while observations were taking place from a 5 metre motor vessel. The individual killer whales and their genealogies were identified using Towers *et al*.^30^. Data on age, sex, associations and observations from other encounters were obtained from the database on this population maintained for the Pacific Biological Station (Fisheries and Oceans Canada).

## Electronic supplementary material

Supplementary Discussion S1

Table S1

Movie S1

Movie S2

Movie S3

Recording S1

Recording S2
