# Author Correction: Infanticide in a mammal-eating killer whale population

**DOI:** 10.1038/s41598-022-10141-y

**Published:** 2022-04-19

**Authors:** Jared R. Towers, Muriel J. Hallé, Helena K. Symonds, Gary J. Sutton, Alexandra B. Morton, Paul Spong, James P. Borrowman, John K. B. Ford

**Affiliations:** 1 Pacific Biological Station, Fisheries and Oceans Canada, 3190 Hammond Bay Road, Nanaimo, BC V9T 6N7 Canada; 2 Bay Cetology, Box 554, Alert Bay, BC V0N 1A0 Canada; 3 OrcaLab, Pacific Orca Society, Box 510, Alert Bay, BC V0N 1A0 Canada; 4 Raincoast Research Society, Box 399, Sointula, BC V0N 3E0 Canada; 5 Whale Interpretive Centre, Box 2–3, Telegraph Cove, BC V0N 3J0 Canada

Correction to: *Scientific Reports* 10.1038/s41598-018-22714-x, published online 20 March 2018

This Article contains an error in Figure 2 where the sex of juvenile T046B4 is incorrect and the sex of juvenile T046B1A is listed as unknown has since been determined. The correct Figure 2 and accompanying legend appear below.

**Figure 2:**
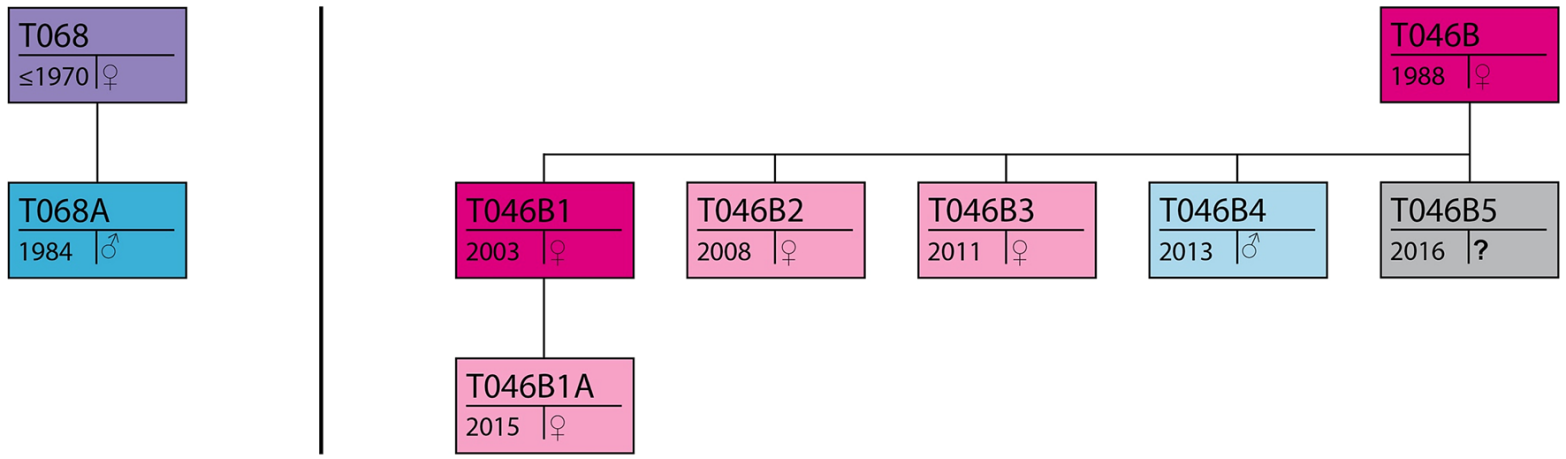
Geneological schematic of present individuals. The identities and where known, sex and birth year of all whales present and their maternal relations to each other (see original schematics in Towers et al*.*^30^) displayed in colour coded statuses. Purple: post-reproductive female, Blue: reproductive male, Fuscia: reproductive females, Pink: juvenile females, Light Blue: juvenile male, Gray: neonate of unknown gender.

As a result, in the Results section,

“Adult female T068 (age ≥ 46 yr, thus post-reproductive) and her adult son T068A (age 32) were travelling together about 200 m behind a young mother T046B1 (age 13), her offspring T046B1A (age < 2 yr) and her sister T046B4 (age < 3 yr) (Fig. 2).”

should read:

“Adult female T068 (age ≥ 46 yr, thus post-reproductive) and her adult son T068A (age 32) were travelling together about 200 m behind a young mother T046B1 (age 13), her daughter T046B1A (age < 2 yr) and her brother T046B4 (age < 3 yr) (Fig. 2).”

