# Supplementary material for: Infanticide in a mammal-eating killer whale population
Source: Sci Rep. 2018 Mar 20;8:4366. doi: 10.1038/s41598-018-22714-x (PMC5861072; doi:10.1038/s41598-018-22714-x)
Supplement: Supplementary file 1 — Supplementary Discussion S1 [file 41598_2018_22714_MOESM1_ESM.pdf]

Supplementary Information

Article in *Scientific Reports*

Infanticide in a mammal-eating killer whale population

Jared R. Towers, Muriel J. Hallé, Helena K. Symonds, Gary J. Sutton, Alexandra B. Morton, Paul Spong, James P. Borrowman, John K.B. Ford

Supplementary Discussion S1

Implications of sexually selected infanticide in killer whales As the most extreme behavioural trait of sexual coercion[1] and result of sexual conflict, infanticide is thought to be a key driver fuelling arms races between the sexes, thereby resulting in the evolution of sex specific morphological traits[2-4]. In turn, these traits can be good indicators of male mating strategies. For example, large size in male odontocetes may be selected in physical competitions with other males (contest competition), for speed and agility to access females (scramble competition) and/or as a display to influence female choice[5]. Killer whales are highly sexually dimorphic with males up to 17% longer and 40% heavier than females. However, morphometric features and measurements differ between the sexes of distinct populations[6-8], some of which have been proposed as distinct species[9]. This may be a sign that sexual conflict contributes to speciation as predicted by Rice[10]. However, if these and any observed differences in scarring rates are an indication of the degree of sexual conflict represented by different populations, it is then possible that their mating systems differ such as they do with morphologically distinct populations of spinner dolphin in the eastern Pacific[11]. Although the mating strategies of most odontocetes are largely unknown, mammal species with large testes relative to body size typically utilize sperm competition as a mating strategy[5]. Large testes size is found in most terrestrial mammal species known to commit infanticide[12]. It is also an indicator of promiscuity or polyandry[13-14] – common mating strategies used by females to confuse paternity and therefore reduce risk of infanticide by males[15-17]. Killer whale males have large testes relative to body size[18] but as females are well known for being pod leaders, Baird[19] indicated that reproductive females in the WCT population may exercise choice by deciding which unrelated adult males are allowed to travel with them. Similarly, Barrett-Lennard[20] suggested that the absence of intra-pod matings in the northern resident killer whale population indicated that females exercise mate choice. On the other hand, our observations of infanticide indicate that morphological traits found only in male killer whales may be sexually selected for more reasons than influencing female choice and that the extent of mate choice by females in the WCT population may also be determined by the risk of coercion directed by unrelated males. If so, it is possible that killer whales may utilize a combination of mating strategies depending on the situation.

References

1. Smuts, B. B. & Smuts, R. W. Male aggression and sexual coercion of females in nonhuman primates and other mammals: evidence and theoretical implications In *Advances in the Study of Behaviour: Volume 22*, (eds. Slater, P., Milinski, M., Snowden, C. & Rosenblatt, J.) 1-63 (Academic Press, 1993).

2. van Schaik, C. P. & Janson, C. H. Infanticide by males: prospectus In *Infanticide by Males and its Implications,* (eds. van Schaik, C. P. & Janson, C. H.) 1-6 (Cambridge Univ. Press, 2000).

3. Parker, G. A. Sexual selection and sexual conflict In *Sexual Selection and Reproductive Competition in Insects*, (eds. Blum, M. S. & Blum, N. A.) 123-166 (Academic Press, 1979).

4. Parker, G. A. Arms races in evolution: An ESS to the opponent-independent costs game. *J. Theor. Biol.* **101**, 619-648 (1983).

5. Boness, D. J., Clapham, P. J. & Mesnick, S. L. Life history and reproductive strategies In *Marine Mammal Biology: An Evolutionary Approach*, (ed. Hoelzel A. R.) 278-324 (Blackwell Science Ltd, 2002).

6. Ford, J. K. B. *Marine Mammals of British Columbia* (Royal BC Museum, 2014).

7. Pitman, R. L. & Ensor, P. Three forms of killer whales (*Orcinus orca*) in Antarctic waters. *J. Cetacean Res. Manage*. **5**, 131-139 (2003).

8. Durban, J. W., Fearnbach, H., Burrows, D. G., Ylitalo, G. M. & Pitman, R. L. Morphological and ecological evidence for two sympatric forms of Type B killer whale around the Antarctic Peninsula. *Polar Biol.* **40**, 231-236 (2017).

9. Morin, P. A. *et al*. Complete mitochondrial genome phylogeographic analysis of killer whales (*Orcinus orca*) indicates multiple species. *Genome Res.* **20**, 908-916 (2010).

10. Rice, W. R. Sexually antagonistic male adaptation triggered by experimental arrest of female evolution. *Nature* **381**, 232-234 (1996).

11. Perrin, W. F. & Mesnick, S. L. Sexual ecology of the spinner dolphin, *Stenella longirostris*: Geographic variation in mating system. *Mar. Mamm. Sci.* **19**, 462-483 (2003).

12. Lukas, D. & Huchard, E. Sexual conflict: The evolution of infanticide by males in mammalian societies. *Science* **346,** 841-844 (2014).

13. Parker, G. A. Sperm competition and its evolutionary consequences in the insects. *Biol. Rev*. **45**, 525-567 (1970).

14. Gomendio, M., Harcourt, A. H. & Roldan, E. R. S. Sperm competition in mammals In *Sperm Competition and Sexual Selection,* (eds. Birkhead, T. R. & Møller, A. P.) 667–751 (Academic Press, 1998).

15. Agrell, J., Wolff, J. O. & Ylönen, H. Counter-strategies to infanticide in mammals: Costs and consequences. *Oikos* **83**, 507-517 (1998).

16. Wolff, J. O. & MacDonald, D. W. Promiscuous females protect their offspring. *Trends Ecol Evol*. **19**, 127-13 (2004).

17. Klemme, I. & Ylönen, H. Polyandry enhances offspring survival in an infanticidal species. *Biol. Lett.* **6**, 24-26 (2010).

18. Connor, R. C., Read, A. J. & Wrangham, R. Male reproductive strategies and social bonds In *Cetacean Societies: Field Studies of Dolphins and Whales,* (eds. Mann, J., Connor, R. C., Tyack, P. L. & Whitehead, H.) 247-269 (Univ. Chicago Press, 2000).

19. Baird, R. W. The killer whale: foraging specializations and group hunting In *Cetacean Societies: Field*

Studies of Dolphins and Whales, (eds. Mann, J., Connor, R. C., Tyack, P. L. & Whitehead, H.) 127-153 (Univ. Chicago Press, 2000).

20. Barrett-Lennard, L. G. *Population structure and mating patterns of killer whales (Orcinus orca) as revealed by DNA analysis.* PhD thesis, UBC Vancouver (2000).
